# Retraction Note to: Effects of artificial intelligence aibo intervention on alleviating distress and fear in children

**DOI:** 10.1186/s13034-024-00830-z

**Published:** 2024-10-14

**Authors:** Kyoko Tanaka, Maoko Hayakawa, Chihiro Noda, Akio Nakamura, Chieko Akiyama

**Affiliations:** 1 National Center for Child Health and Development, Setagaya-Ku, Tokyo, Japan; 2 Ochanomizu University Human Developmental Sciences, Bunkyo-Ku, Tokyo, Japan; 3 Department of Pediatrics Juntendo University Faculty of Medicine, Bunkyo-Ku, Tokyo, Japan; 4 Clinic for Children, Akiyama Kodomo Clinic, Mitaka, Tokyo Japan; 5 Division of Adolescent Mental Health, Department of Psychosocial Medicine, National Center for Child Health and Development, 2-10-1 Okura, Setagaya- Ku, Tokyo, 157-8535 Japan

**Retraction Note: Child and Adolescent Psychiatry and Mental Health (2022) 16:87** 10.1186/s13034-022-00519-1

The authors have retracted this article because the appropriate ethical review for the study was not conducted. Additionally, the patient consent forms used did not meet the necessary requirements for intervention research.

All authors agree to this retraction.

